# Effects of microplastic types and shapes on the community structure of arbuscular mycorrhizal fungi in different soil types

**DOI:** 10.1007/s11356-025-36408-1

**Published:** 2025-05-02

**Authors:** Daniel R. Lammel, Shin Woong Kim, Lili Rong, Hongyu Chen, Rosolino Ingraffia, Matthias C. Rillig

**Affiliations:** 1 Institut Für Biologie, Freie Universität Berlin, 14195 Berlin, Germany; 2 Berlin-Brandenburg Institute of Advanced Biodiversity Research (BBIB), Berlin, D- 14195 Germany; 3 Center for Ecotoxicology and Environmental Future Research, Korea Institute of Toxicology, 17 Jegok-Gil, Jinju, 52834 Republic of Korea; 4 MOE Key Laboratory of Pollution Processes and Environmental Criteria, College of Environmental Science and Engineering, Nankai University, Tianjin, 300350 China; 5 Department of Agricultural, Food and Forestry Sciences, University of Palermo, Palermo, Italy

**Keywords:** Arbuscular mycorrhiza fungi, Microplastics, Composition, Shapes, Diversity

## Abstract

Arbuscular mycorrhizal fungi (AMF) play a crucial role in soil ecosystems by enhancing nutrient exchange, stabilizing soil structure, and improving water movement in soils. Microplastics (MPs), synthetic polymers smaller than five mm, pose an emerging threat to AMF by altering soil structure and chemistry, potentially disrupting these essential functions. This study examined how ten different microplastic types and shapes influenced AMF communities within the context of Grime’s C-S-R (competitor-stress tolerator-ruderal) framework. We tested the effects of polypropylene, polyester, high-density polyethylene, polyethylene terephthalate, low-density polyethylene, polyvinyl chloride, and polystyrene on three distinct soils (Albic Luvisol, Haplic Chernozem, and Haplic Luvisol), examining MPs in forms of fibers, films, fragments, and mixtures, in a total of 15 treatments. Although general diversity indices based on the OTU level showed no significant changes, shifts occurred at the genus level. The soils were predominantly colonized by *Funneliformis* and an unidentified genus in *Glomeraceae (incertae sedis)*; however, the addition of MPs increased the relative abundance of *Diversispora* and *Claroideoglomus*, while also subtly promoting *Rhizophagus* and *Septoglomus*. In particular, *Claroideoglomus* showed a notable increase in relative abundance in the most diverse Haplic Chernozem soil when exposed to fragment-shaped MPs and mixed MP types. According to Grime’s C-S-R framework, these genera are classified as ruderals, except for *Diversispora*, a stress tolerator. Ruderals are known to thrive following disturbances, such as physical disruption of soil structure, a known effect of MPs, while MPs can also create stress conditions within the soil selecting stress tolerators. Our findings highlight the complex and soil-specific interactions between MPs and AMF, demonstrating that the effects of MPs on AMF are driven by the combined influence of soil types and MP properties.

**Supplementary Information:**

The online version contains supplementary material available at 10.1007/s11356-025-36408-1.

## Introduction

Arbuscular mycorrhizal fungi (AMF) are soil fungi that form mutualistic associations with plants, absorbing and exchanging essential nutrients, such as phosphorus, for carbon compounds derived from plants. This symbiosis is crucial for terrestrial ecosystems, as AMF enhance soil structure, stabilize aggregates through hyphal networks, and improve water and nutrient movement (Leifheit et al. 2021). However, microplastics (MPs), synthetic polymers under 5 mm in diameter, present an emerging threat to AMF by potentially disrupting AMF functions and modifying soil structure and chemistry (Machado et al. 2019; Leifheit et al. 2021).

MPs are diverse in origin and composition and are predominantly generated and used in industrial and urban environments, but also enter soils and agricultural environments through various means, including as atmospheric deposition, as tire wear particles from roadways, in sewage sludge and from plastic littering (Leifheit et al. 2021; Chen et al. 2024; Sahai et al. 2025). MPs can also contaminate agriculturally cultivated soils in additional ways, including contaminated compost additions, plastic mulch, contaminated irrigation water, polymer-coated fertilizers, and tools and machines which contain or are painted with MPs (Leifheit et al. 2021; Chen et al. 2024; Sahai et al. 2025). Common MP polymers include polypropylene (PP), polyester (PES), polyethylene (PE), at low (LDPE) and high densities, polyethylene terephthalate (PET), polyvinyl chloride (PVC), and polystyrene (PS). Each polymer exhibits different physicochemical properties, and thus having unique degradation rates, and influencing soil structure, biota, water retention, and nutrient cycling differently (Machado et al. 2019; Leifheit et al. 2021; Chen et al. 2024). Also, not only MP composition, but also shapes, such fibers, fragments, and films, exert distinct impacts on soil ecology (Lozano et al. 2021). For example, PES and PP fibers, common in textiles, can block soil pore spaces, affecting porosity, water transport, and microbial access. HDPE and LDPE fragments and films can compact soil, affecting bulk density and water-holding capacity (Wan et al. 2019). Fibers can destabilize soil aggregates, while fragments like PET, PVC, or PS can embed within soil particles, influencing aeration and microbial interactions (Machado et al. 2019; Lozano et al. 2021).

The influence of MP types and shapes on AMF are potentially complex (Leifheit et al. 2021). Fibrous MPs, such as PES or PP, can alter soil bulk density and aggregate stability, impacting hyphal growth depending on soil type and pore structure. Fibers may increase pore size and water availability but obstruct root access, disturbing AMF colonization by blocking fine roots. In contrast, PET and HDPE fragments potentially alter soil texture and pore connectivity, affecting AMF’s ability to penetrate soil and exchange nutrients (Machado et al. 2019; Leifheit et al. 2021). Kanold et al. (2024) suggested that polyester MP fibers, due to their hydrophilicity, could enhance soil water-holding capacity; however, increased MP accumulation could impair AMF by altering porosity, bulk density, and soil chemistry, affecting nutrient cycling and AMF-plant interactions (Leifheit et al. 2021; Kanold et al. 2024).

AMF community composition, defined by the diversity and relative abundance of AMF taxa in soil, is vital for maintaining ecosystem processes (Leifheit et al. 2021). Changes in AMF composition could impact nutrient cycling, soil aggregation, and plant resilience, with MPs contributing to shifts in AMF communities based on the different genera’s response to the contaminants (Leifheit et al. 2021).

A few studies have tested the effects of MPs and biodegradable polylactic acid (PLA), mostly combined with heavy metals, on AMF. Wang et al. (2020) demonstrated that both polyethylene (PE) and biodegradable PLA affected AMF diversity, with outcomes varying by MP type and concentration. Only PLA at a 1% concentration, compared to the control, increased AMF richness and the Shannon index. However, PE doses increased the relative abundance of an unclassified *Glomeraceae* genus, while PLA reduced it. There was a relative increase in *Ambispora* and an *Archaeosporaceae* with some PLA and PE additions. Yang et al. (2021) further reported that high doses of HDPE and PLA little impacted AMF diversity indices, but a reduction of relative abundance of *Glomus* was observed for HDPE addition and an increase of *Archaesopora* for PLA addition. Furthermore, Liu et al. (2023) reported that PET, PLA, and PES also had little influenced on AMF diversity indices, and only a high concentration of PLA (2%) reduced the Shannon index. *Glomus* dominated all treatments, and only a reduction of relative abundance of *Acaulospora* and *Paraglomus* in 2% PLA was evident. In contrast, Wei et al. (2024) saw no effect of PLA and PE on AMF diversity. Moreover, Kanold et al. (2024) provided further insights into the impact of PE fibers, finding that very high concentrations (3%) favor competitive taxa such as *Gigaspora* while reducing ruderal species like *Glomus*. Lastly, Shirin et al. (2024) reported that PS of different sizes decreased the relative abundance of *Glomeraceae*, and 100 µm PS increased *Diversisporaceae*, while the combination with heavy metals increased *Paraglomeraceae*.

These pioneer studies demonstrated that the impact of MPs on AMF community structures are soil and polymer type dependent. Overall shifts align with Grime’s competitor-stress tolerator-ruderal (C-S-R) framework applied to AMF (Chagnon et al. 2013; Kanold et al. 2024). In this framework, competitive AMF (C, such as *Gigasporaceae*) thrive in stable, enriched environments, while stress-tolerant taxa (S, such as *Acaulosporaceae*), are adapted to low-resource environments, while ruderal (R, such as *Glomeraceae*), characterized by rapid colonization and high reproductive rates, can flourish under disturbed conditions. The previous studies suggested that usually MP-mediated stress on soil, such as biodegradation of PLA, increase the relative abundance of ancestral, likely stress tolerator, taxa (e.g., *Archaeosporaceae*, *Diversisporaceae*), while other MPs, such as PE fibers, create conditions that benefit competitive AMF taxa (e.g., *Gigaspora*) (Chagnon et al. 2013; Cahyaningtyas and Ezawa 2024). Thus, MPs appear to selectively influence AMF composition by fostering conditions selecting for different growth strategy traits.

Given that these previous studies demonstrated that AMF responses depended on both MP types and soil origins, and were limited to only a few polymer types, our objective in this study was to investigate how ten different soil polymer types, shapes, and combinations affect AMF community structure.

The study was based on two hypotheses. First, we hypothesize that different MP polymers and shapes create soil conditions influencing AMF composition within the C-S-R framework. Competitive AMF (e.g., *Gigasporaceae*) may thrive with PES fibers that increase water retention, fostering enriched, stable environments. Stress-tolerant AMF (e.g., *Acaulosporaceae*) should be favored by fragment and film MPs that create stressed, compacted habitats, while ruderal taxa (e.g., *Glomeraceae*) are likely reduced, because fine pores can be blocked limiting resource access and connectivity. Our second hypothesis posits that exposure to combinations of MPs with different polymers and shapes exert compounded selective pressures on AMF communities, intensifying shifts in composition beyond those induced by single MP types. In mixed-MP environments, we expect intensified selective pressures that could simplify AMF community structure, potentially favoring stress-tolerant taxa while reducing competitive and ruderal taxa.

## Material and methods

### Soil origin and experimental setup

This study was built on a previous experiment which tested the effects of different types of polymers, shapes, and combinations on three different soils (Chen et al. 2024). Three agricultural soil types were sampled from the top layer (< 30 cm depth) of fields located at separate experimental sites across Germany. According to FAO soil classification, these soils included the following: Albic Luvisol (soil A), a loamy sand soil; Haplic Chernozem (soil B), with a silty loam texture; and Haplic Luvisol (soil C), also a silty loam. Soil characteristics are provided in Supp. Table 1.

Each soil was divided into 15 treatments for each soil type, including one control, 10 plastic types of various polymers and/or shapes, and four plastic mixtures. Control soils had 16 replicates, single MP treatments had 8 replicates, and mixtures had 10 replicates. The experiment included a total of 408 experimental units, which were pots filled with 0.7 L of soil each. Soils were equally treated with 0.4% w/v of different MPs (zero for controls), and ensuring good homogenization and no cross-contamination (for details, see Chen et al. 2024).

The 10 single MP treatments were as follows: fiber HDPE, fiber PES, fiber PP, film LDPE, film PET, film PP, film PVC, fragment PP, fragment PE, and fragment PVC. MP characteristics are provided in Supp. Table 2. The four MP mixture treatments were prepared following the random sampling approach from pools (Brennan and Collins 2015; Chen et al. 2024). In this setup, MP types (or “factors”) were randomly chosen from the pool of 10 single treatments to create different replicates with a specified number of MPs. This approach enables assessing the effects of an increasing number of MP sources in combination and deriving general insights into how variations in MP diversity impact soil responses, independently of individual MP identities. The mixtures were composed of 2, 5, 8, and 10 combinations of the MP treatments. Each replicate of each mixture was composed of a random selection from the 10 MPs, added to the soil while maintaining the same final concentrations (for additional details, see Chen et al. 2024).

Pots with soil were then irrigated to field capacity water content and incubated in a greenhouse for eight weeks with a temperature of 20 °C. After this incubation time, each pot received two wheat seeds, and after germination, all were thinned to one plant per pot. All handling was done with sterile material to avoid cross contamination. Wheat was chosen as the host plant as it represents a commonly cultivated crop in the region where the soils were collected, and 20 °C approximates the average temperature of these regions during the crop season. Wheat is known to be colonized by AMF; thus, root colonization was not evaluated in this study, as our aim was to analyze the effects on the extraradical AMF directly exposed to MP in the soil. Soils were watered in relation to the controls. Controls were weighted twice a week and watered to achieve 60% of field capacity. The same amount of water was added for the different treatments of each soil. Pots were fertilized three times with 25 mg of N as ammonium nitrate (equivalent to 138 kg of N per ha), at 5, 10, and 15 days after emergence. The experiment was harvested after 8 weeks, the soil was separated from the roots, and aliquots frozen in 2-mL conical tubes until DNA extraction (Chen et al. 2024).

### Molecular characterization of the AMF communities

DNA was extracted from 250 mg of soil using the PowerSoil DNA isolation kit (QIAGEN). PCR amplification followed the method by Roy et al. (2017). In a first step, DNA was amplified using the nested-PCR covering the ITS-LSU region of AMF, as proposed by Krüger et al. (2009). Later, a nested-PCR amplified the D1–D2 region of the rDNA large subunit (LSU) using universal fungal primers LR2rev and LR3 (Roy et al. 2017). Last, samples were tagged with an 8 nt index for sample multiplexing. The final PCR products were purified, pooled at equimolar concentrations, and sequenced on Illumina MiSeq 2 × 300 base paired-end at the Berlin Center for Genomics in Biodiversity Research (BeGenDiv). Sequences were deposited in NCBI GenBank under BioProject PRJNA1230207.

Sequences were processed using Usearch 11 (https://drive5.com/usearch). The reads were quality filtered, primers trimmed out, denoised, chimera filtered, and clustered into operational taxonomic units (OTUs) at 97% similarity (Edgar 2013). Reads were then mapped to the OTUs to generate the counting table for each sample. The tables were rarefied to the same sequencing depth, allowing a fair comparison among samples. The OTU sequences were annotated using a Naïve Bayesian classifier and the RDP LSU database (Wang et al. 2007). AMF taxonomy remains an ongoing debate, with no consensus on species definition based on short ribosomal sequences. Only a few AMF genomes have been sequenced, and species thresholds are still not well established, while efforts to reconcile genome-based classification with traditional spore morphology have only recently emerged (Oliveira et al. 2024). Since this study relied on short LSU sequences of unknown environmental taxa, we adopted a conservative approach by identifying OTUs at the genus level (Tedersoo et al. 2020).

### Statistics

Analyses were performed in R v.4.4.1 (R Core Team 2024). First, the package Vegan was used to calculate alpha and beta-diversity. Richness and Shannon diversity were calculated and samples compared by one and two ways analysis of variance (ANOVA). Beta-diversity was evaluated by comparing Bray–Curtis dissimilarity matrices using perMANOVA, as also samples were ordinated by a principal coordinates analysis (PCoA). Since sequencing data is compositional, the relative abundance of the AMF taxa was analyzed using the Analysis of Compositions of Microbiomes with Bias Correction (ANCOM-BC), which was specifically developed to handle amplicon sequencing data (Lin et al. 2020).

## Results

Three different soils were evaluated for AMF diversity using amplicon sequencing of the LSU region. We recovered an average of 5.2 thousand sequences per sample, which varied in proportion of AMF according to soil types (Supp. Figure 1). The Haplic Chernozem had the highest proportion of AMF sequences (73%), followed by Albic Luvisol (38%) and Haplic Luvisol (only 0–25%). Some samples of the Haplic Luvisol soil did not yield AMF sequences and were removed. All samples were then rarefied to one thousand reads, allowing equal comparison of the samples.

A total of 72 AMF OTUs (Supp. Figure 2) were identified across the samples and soils, belonging to three families and eight genera, respectively, *Claroideoglomeraceae* (*Claroideoglomus*), *Diversisporaceae* (*Diversispora* and *Diversispora* incertae sedis), and *Glomeraceae* (*Funneliformis*, *Glomus*, *Glomeraceae* incertae sedis, *Rhizophagus*, and *Septoglomus*). Since the analyses were based on environmental sequences, two of the genera could not be identified in formal taxonomy, but rather predicted by the sequence classifier tool as gen. “*incertae sedis*.”

**Fig. 1:**
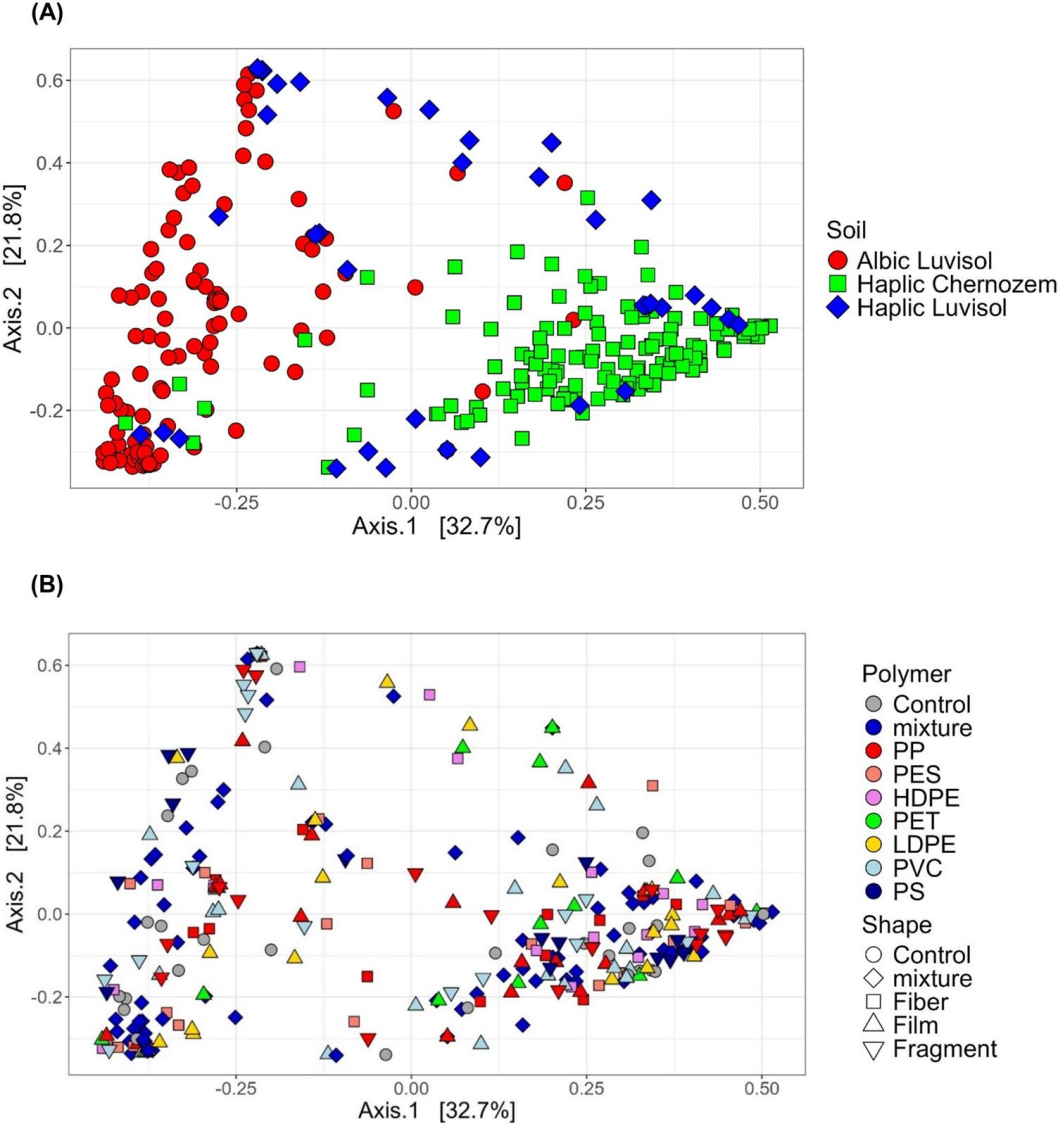
Beta-diversity expressed as a PCoA ordination based on Bray–Curtis dissimilarity. Samples are identified by soil sample (**A**) or by polymer type and shape (**B**). Polymers are polypropylene (PP), polyester (PES), high-density polyethylene (HDPE), polyethylene terephthalate (PET), low-density polyethylene (LDPE), polyvinyl chloride (PVC), and polystyrene (PS) and mixture of microplastics (for details, see the “Methods” section)

**Fig. 2:**
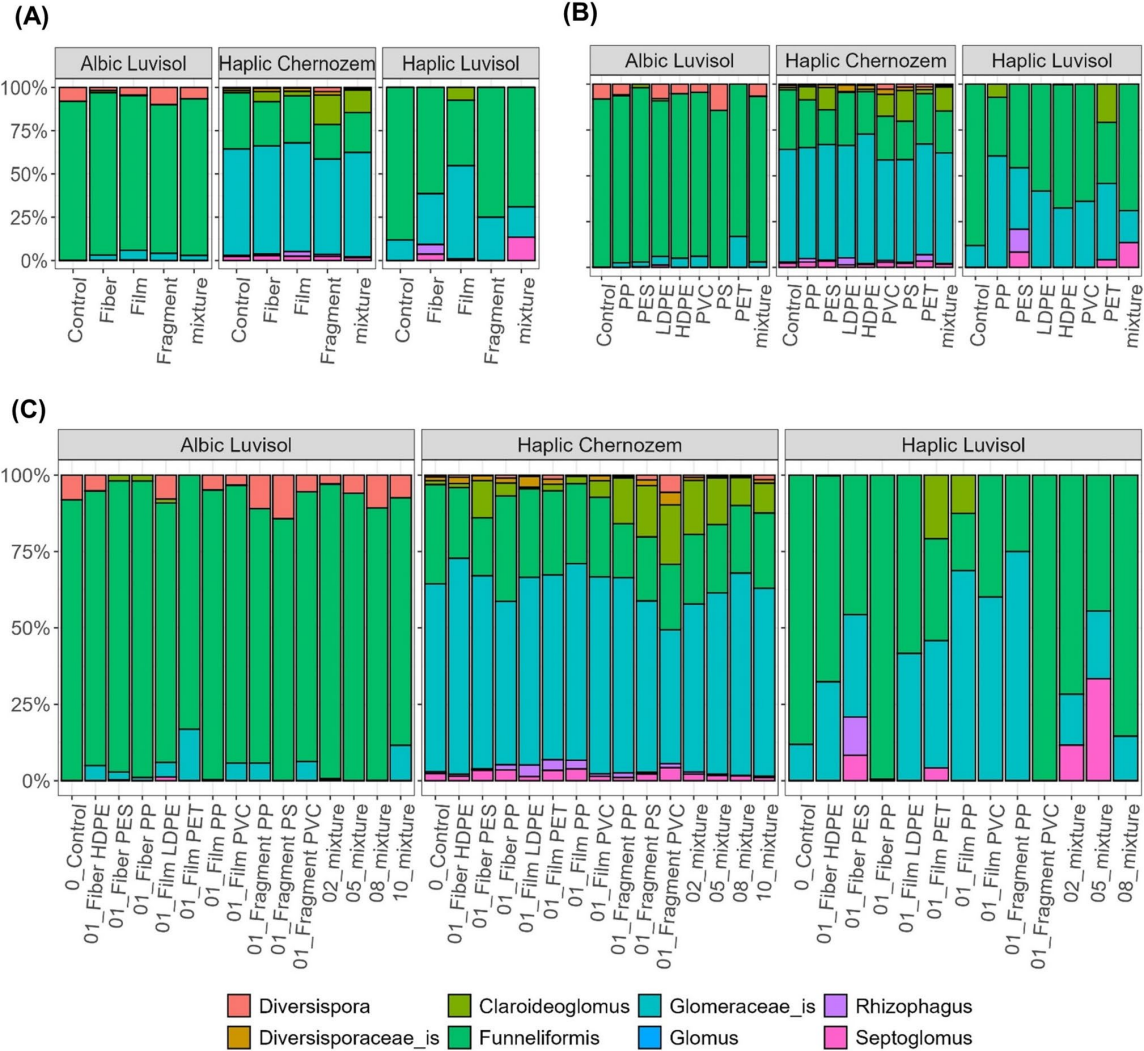
Panel depicting the relative abundance of AMF across treatments for each soil type, averaged by microplastic shape (**A**), polymer type (**B**), and all combinations individually (**C**). Polymers are polypropylene (PP), polyester (PES), high-density polyethylene (HDPE), polyethylene terephthalate (PET), low-density polyethylene (LDPE), polyvinyl chloride (PVC), and polystyrene (PS). The levels 02, 05, 08, and 10 refer to mixtures of microplastics (for details see, the “Methods” section)

Alpha and beta diversity indices showed a clearly evident difference among the different soils, but not among the treatments (Supp. Figure 2 and 3 for OTU richness and Shannon diversity index, and Fig. 1 for beta diversity). The Haplic Chernozem was clearly the most diverse, presenting the highest richness of AMF, ranging from 4 to 32 OTUs across samples, while the other soils ranged between 0 and 11 OTUs per sample (Supp. Figure 2 A–C). Beta diversity analysis, represented by PCoA ordination of Bray–Curtis dissimilarities across samples (Fig. 1A) and genus composition (Fig. 2A), indicated that the Albic Luvisol and Haplic Chernozem were distinctly different, whereas the Haplic Luvisol exhibited an intermediate composition between them (Fig. 1A and Supp. Figure 2).

**Fig. 3:**
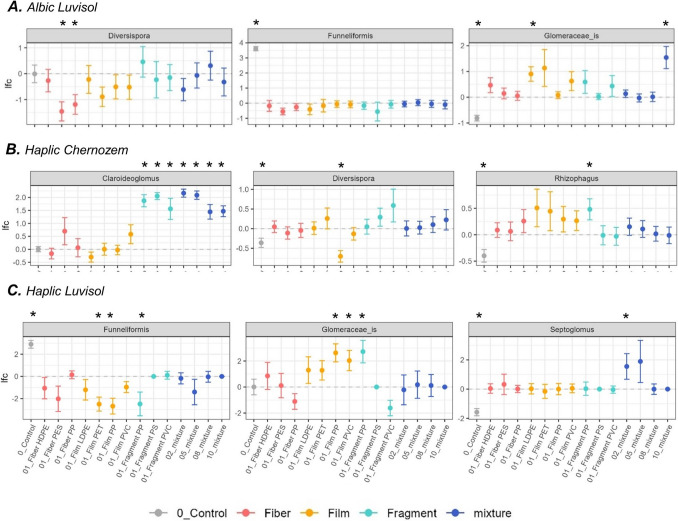
Panel depicting shifts in the relative abundance of AMF genera across treatments for each soil type. The dotted line represents the ANCOM-BC baseline across treatments, with the log-fold change (lfc) for each treatment plotted relative to the baseline as mean values with standard error. Statistically significant differences (*p* < 0.05) are marked with an asterisk. The panel is organized by genus and soil type: Albic Luvisol (**A**), Haplic Chernozem (**B**), and Haplic Luvisol (**C**). Polymers include polypropylene (PP), polyester (PES), high-density polyethylene (HDPE), polyethylene terephthalate (PET), low-density polyethylene (LDPE), polyvinyl chloride (PVC), and polystyrene (PS). The levels 00, 01, 02, 05, 08, and 10 refer to the number of microplastics on each mixture (details in the “Methods” section)

All soils were primarily dominated by the genus *Funneliformis* and a genus from *Glomeraceae* incertae sedis (_is) (Fig. 2). Following the application of MPs, effects at the genus level were detected (Fig. 3, Supp. Figure 4). Specifically, in the Albic Luvisol soil, PES and PP fibers led to a decrease in the relative abundance of *Diversispora* compared to the control. Conversely, *Funneliformis* was most abundant in the control, whereas *Glomeraceae_is* exhibited the lowest abundance in the control but increased significantly in the presence of PET films and 10-plastic mixtures (Fig. 3A). In the Haplic Chernozem soil, *Claroideoglomus* notably increased with fragment-shaped MPs and mixtures, while MPs increased *Diversispora* and *Rhizophagus* relative to control (Fig. 3B). In the Haplic Luvisol, MP additions reduced *Funneliformis* relative abundances, by increasing *Glomeraceae_is*, and subtly promoting *Septoglomus* (Fig. 3C).

**Fig. 4:**
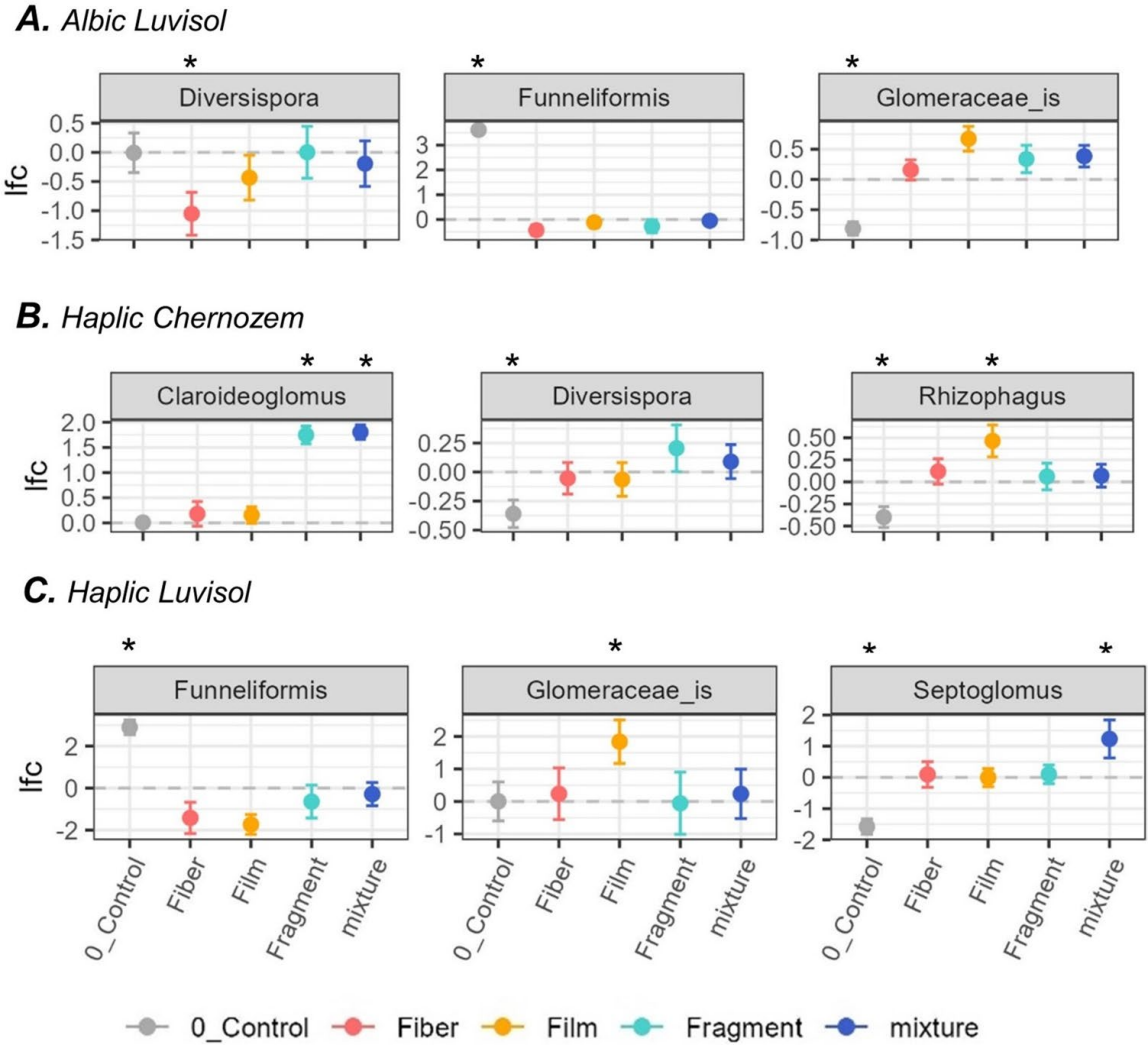
Panel depicting shifts in the relative abundance of AMF genera across microplastic shapes for each soil type. The dotted line represents the ANCOM-BC baseline across treatments, with the log-fold change (lfc) for each treatment plotted relative to the baseline as mean values with standard error. Statistically significant differences (*p* < 0.05) are marked with an asterisk. The panel is organized by genus and soil type: Albic Luvisol (**A**), Haplic Chernozem (**B**), and Haplic Luvisol (**C**). Polymers include polypropylene (PP), polyester (PES), high-density polyethylene (HDPE), polyethylene terephthalate (PET), low-density polyethylene (LDPE), polyvinyl chloride (PVC), polystyrene (PS), and polymer mixture (details in the “Methods” section)

Aggregating data at the polymer shape level revealed eight differences (Fig. 4). In Albic Luvisol, fiber-shaped MPs reduced *Diversispora* relative abundance, while the control was dominated by *Funneliformis* and had reduced *Glomeraceae_is* (Fig. 4A). In Haplic Chernozem, *Claroideoglomus* showed a notable increase with fragment and mixture treatments, and *Rhizophagus* subtly increased with film MPs (Fig. 4B). In Haplic Luvisol, *Funneliformis* dominated the control, while *Glomeraceae_is* increased with film-shaped MPs, and *Septoglomus* was slightly higher in MP mixtures but reduced in the control (Fig. 4C).

## Discussion

Microplastics (MPs) are increasingly recognized as a significant environmental factor impacting soil ecosystems, particularly through interactions with soil microbiota, including AMF (Leifheit et al. 2021). MPs can alter soil properties and create conditions that potentially affect AMF taxa (Wang et al. 2020; Liu et al. 2023; Kanold et al. 2024). This study aimed to investigate these dynamics, specifically examining how different MP polymers and shapes affect AMF community structure through Grime’s C-S-R framework. We hypothesized that MP properties could create distinct soil environments, selectively benefiting competitive and stress-tolerator AMF taxa according to microplastics types. Additionally, we proposed that exposure to combinations of MP types would amplify selective pressures on AMF composition beyond those caused by individual MP exposures.

Our results showed that the AMF communities in all soils and treatments were primarily dominated by *Funneliformis* and a *Glomeraceae_is*, consistent with previous reports that these taxa are responsive to nitrogen additions and agricultural settings, such as our experimental conditions (Treseder et al. 2018). However, with MP additions, the relative abundance of *Diversispora* and *Claroideoglomus* increased, alongside nuanced increases in *Rhizophagus* and *Septoglomus*. This genus-level shift, particularly with *Claroideoglomus* (Claroideoglomeraceae) and *Rhizophagus* and *Septoglomus* (Glomeraceae), suggests a trend toward ruderal taxa, which are typically well suited to disturbed environments (Chagnon et al. 2013; Weber et al. 2019; Horsch et al. 2023). An exception to the ruderal dominance was observed with *Diversispora*, which increased in relative abundance under specific soil-MP combinations involving fragment shapes and mixtures. *Diversispora*, identified as a slow regenerator and edaphophilic (Weber et al. 2019; Cahyaningtyas et al. 2024), is noted for its preference for stable, less disturbed soils, aligning it with a stress-tolerant strategy rather than a ruderal one (Chagnon et al. 2013).

These findings contrast with our initial hypothesis and previous studies, which suggested that predominantly MP exerted stress on soil increased the relative abundance of predominantly stress tolerator taxa (e.g., *Archaeosporaceae*, *Diversisporaceae*). However, most previous studies have involved biodegradable PLA combined with heavy metals, and it is likely that PLA decomposition could have generated soil metabolic stress, selecting for stress-tolerant organisms in those studies (Wang et al. 2020; Yang et al. 2021; Liu et al 2023; Shirin et al. 2024).

On the other hand, the predominance of ruderal taxa in our study is supported by the fact the ruderal trait was also proposed to respond to physical disruption of soil structure (Chagnon et al. 2013), since taxa with high growth rates could cope better with soil structure changes. MPs can change soil structure aggregation and pores (Machado et al. 2019), though likely selecting for this trait.

Nevertheless, our data also showed large variation in the response of the genera to different soil types and to the different MPs, making it difficult to establish specific mechanisms. Since no taxa shifts were observed consistently across all three soils, we cannot assume specific effects of different MPs on taxa. Additionally, it is important to emphasize that sequencing data is compositional (Lin et al. 2020), meaning that increases or decreases in abundance are relative to other taxa within each sample. For instance, the observed reduction in the relative abundance of *Funneliformis* in MP treatments is likely not an absolute decrease but rather a relative shift, as other genera thrived under these conditions, thereby comparatively reducing *Funneliformis* relative abundance. Absolute quantification might elucidate MP effects more clearly; however, such methodology for AMF sequencing is still in development.

Finally, in our study, the alpha and beta diversity indices did not show significant changes. This finding is consistent with most previous studies, which also did not report significant differences in alpha and beta diversity. However, a few studies that did find differences worked with very high concentrations of microplastics (MPs), up to 10%, or biodegradable PLA types (Yang et al. 2021; Kanold et al. 2024). These contrasting findings indicate that the impact of MPs on AMF diversity may depend strongly on MP concentrations and specific environmental conditions. While high MP concentrations could simplify AMF communities or filter taxa, our study’s lower MP concentration (0.4%, which is a reasonable concentration, expected to occur in several decades with continued MP addition; Chen et al. 2024) likely resulted in more subtle shifts, promoting ruderal responses rather than a community shift toward more diverse taxa across treatments.

These results, together with existing literature, corroborate the idea that the characteristics of microplastics (MPs), types, shapes, and doses, as well as the types of soil, are pivotal in influencing arbuscular mycorrhizal fungi (AMF) communities. This underscores the intricate and soil-specific interactions that exist, emphasizing the importance of both factors in determining the ecological dynamics within these communities.

## Conclusion

This study investigated how different types, shapes, and combinations of microplastics (MPs) affect AMF community structure across three soils, guided by the competitor-stress tolerator-ruderal (C-S-R) framework. While overall alpha and beta diversity indices showed no significant changes, genus-level changes indicated nuanced AMF responses to MP exposure. MP addition generally favored ruderal taxa, as seen with increased relative abundances of *Claroideoglomus*, *Rhizophagus*, and *Septoglomus*, likely responding to the physical disturbance of the soil structure caused by the MP. However, *Diversispora* also shifted in specific soil-MP combinations, suggesting stress-tolerant adaptation. These findings, along with current literature, support the hypothesis that both MP properties and soil types play important roles in shaping AMF communities and highlight complex, soil-dependent interactions.

## Supplementary Information

Below is the link to the electronic supplementary material.

ESM 1 (PDF 3.53 MB)

## Data Availability

The manuscript has associated data in a data repository. NCBI Genbank BioProject PRJNA1230207.
