# Supplementary material for: Effects of microplastic types and shapes on the community structure of arbuscular mycorrhizal fungi in different soil types
Source: Environ Sci Pollut Res Int. 2025 May 2;32(20):12504–12. doi: 10.1007/s11356-025-36408-1 (PMC12098463; doi:10.1007/s11356-025-36408-1)
Supplement: Supplementary file 1 — (PDF 3.53 MB) [file 11356_2025_36408_MOESM1_ESM.pdf]

Effects of Microplastic Types and Shapes on the Community Structure of Arbuscular Mycorrhizal Fungi in Different Soil Types

Daniel R. Lammel[1,2], Shin Woong Kim[1,2,3], Lili Rong[1,2,4], Hongyu Chen[1,2], Rosolino Ingraffia[1,2,5], Matthias C. Rillig[1,2]

[1] Freie Universität Berlin, Institut für Biologie, D-14195 Berlin, Germany
[2] Berlin-Brandenburg Institute of Advanced Biodiversity Research (BBIB), D-14195 Berlin, Germany
[3]Center for Ecotoxicology and Environmental Future Research, Korea Institute of Toxicology, 17 Jegok-gil, Jinju 52834, Republic of Korea
[4] MOE Key Laboratory of Pollution Processes and Environmental Criteria, College of Environmental Science and Engineering, Nankai University, 300350 Tianjin, China
[5] Department of Agricultural, Food and Forestry Sciences, University of Palermo, Palermo, Italy

Supplementary Material

Figures and Tables

(A) Total number of Sequences per soil type
(each dot is one sample)

(B) Sequences of AMF per soil type
(each dot is one sample)

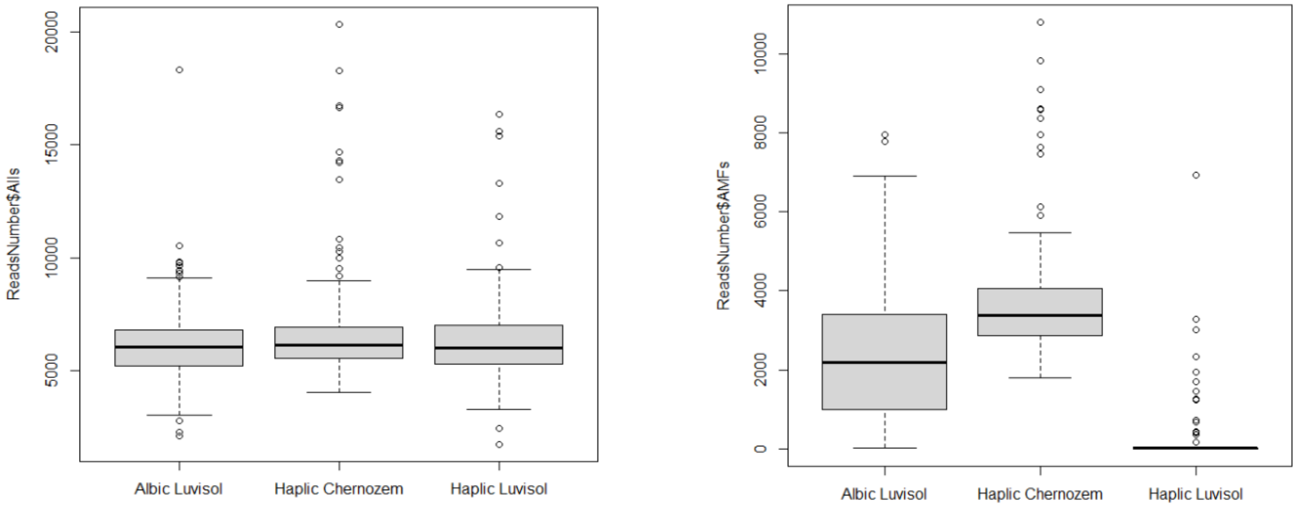

S. Fig. 1 (A) total number of sequences and (B) sequences only of AMF in each sample. There is a drop in relative quantity of sequences of AMF for the Haplic Luisol soil.

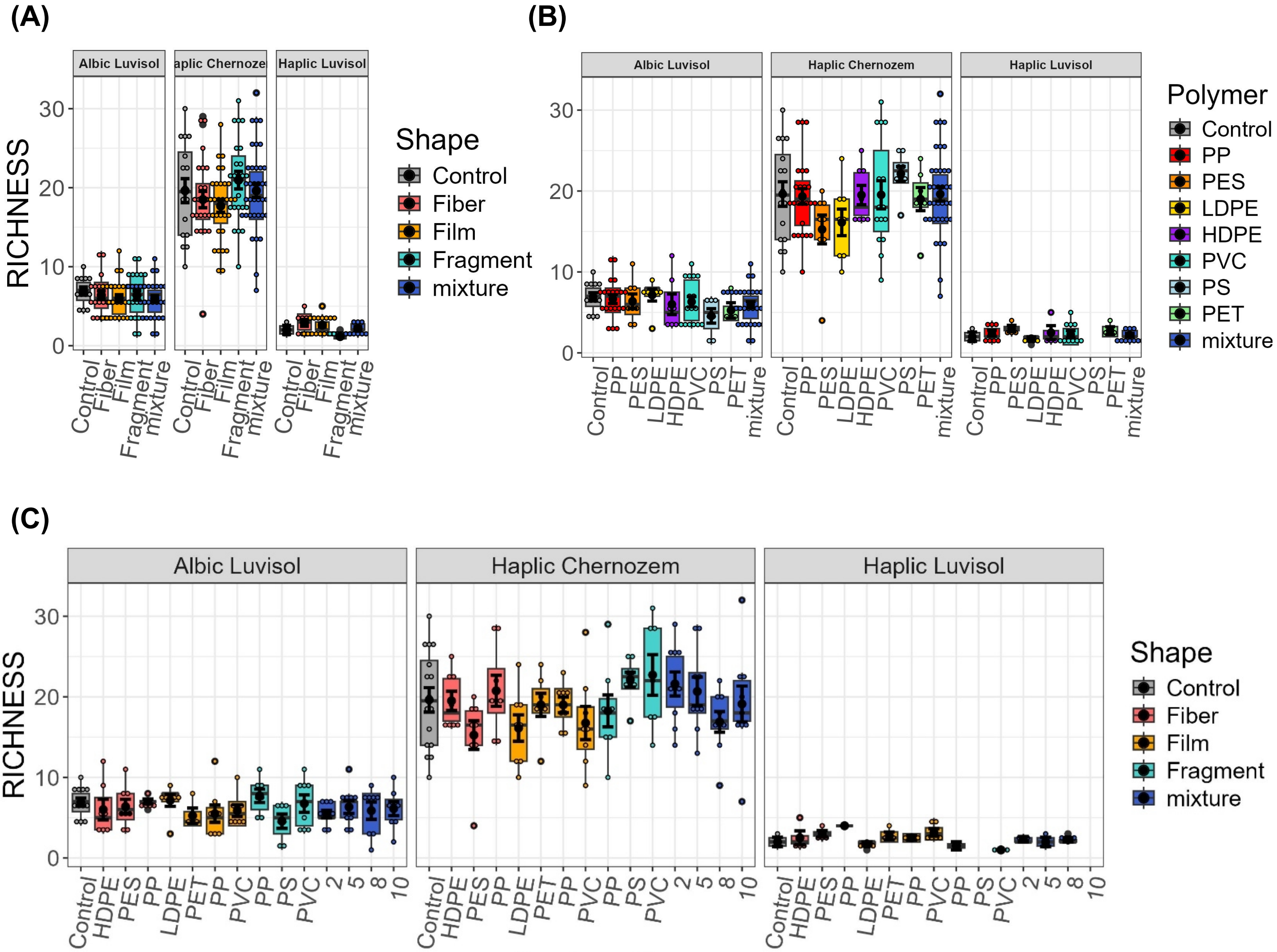

(D)

Albic Luvisol

**One-way ANOVA**	Treatment	***p*=0.43**
**Two-way ANOVA**	Polymer	***p*=0.68**
	Shape	***p*=0.12**
	P:S	***p*=0.32**

Haplic Chernozem

**One-way ANOVA**	Treatment	***p*=0.10**
**Two-way ANOVA**	Polymer	***p*=0.29**
	Shape	***p*=0.16**
	P:S	***p*=0.07**

Haplic Luvisol

**One-way ANOVA**	Treatment	***p*=0.07**
**Two-way ANOVA**	Polymer	***p*=0.18**
	Shape	***p*=0.07**
	P:S	***p*=0.17**

S. Fig. 2 Panel depicting the richness of AMF OTUs in each sample, averaged by micro-plastic shape (**A**), polymer type (**B**) and all combinations labeled by polymer and colored by shape (**C**)**,** and the analysis of variance results (**D**). Black dots are average, error bars are standard error, and gray dots are each individual replicates. Polymers: polypropylene (PP), polyester (PES), high density polyethylene (HDPE), polyethylene terephthalate (PET), low density polyethylene (LDPE), polyvinyl chloride (PVC), and polystyrene (PS). The level 2, 5, 8, and 10 refer to mixtures of microplastics (for details see the Methods section).

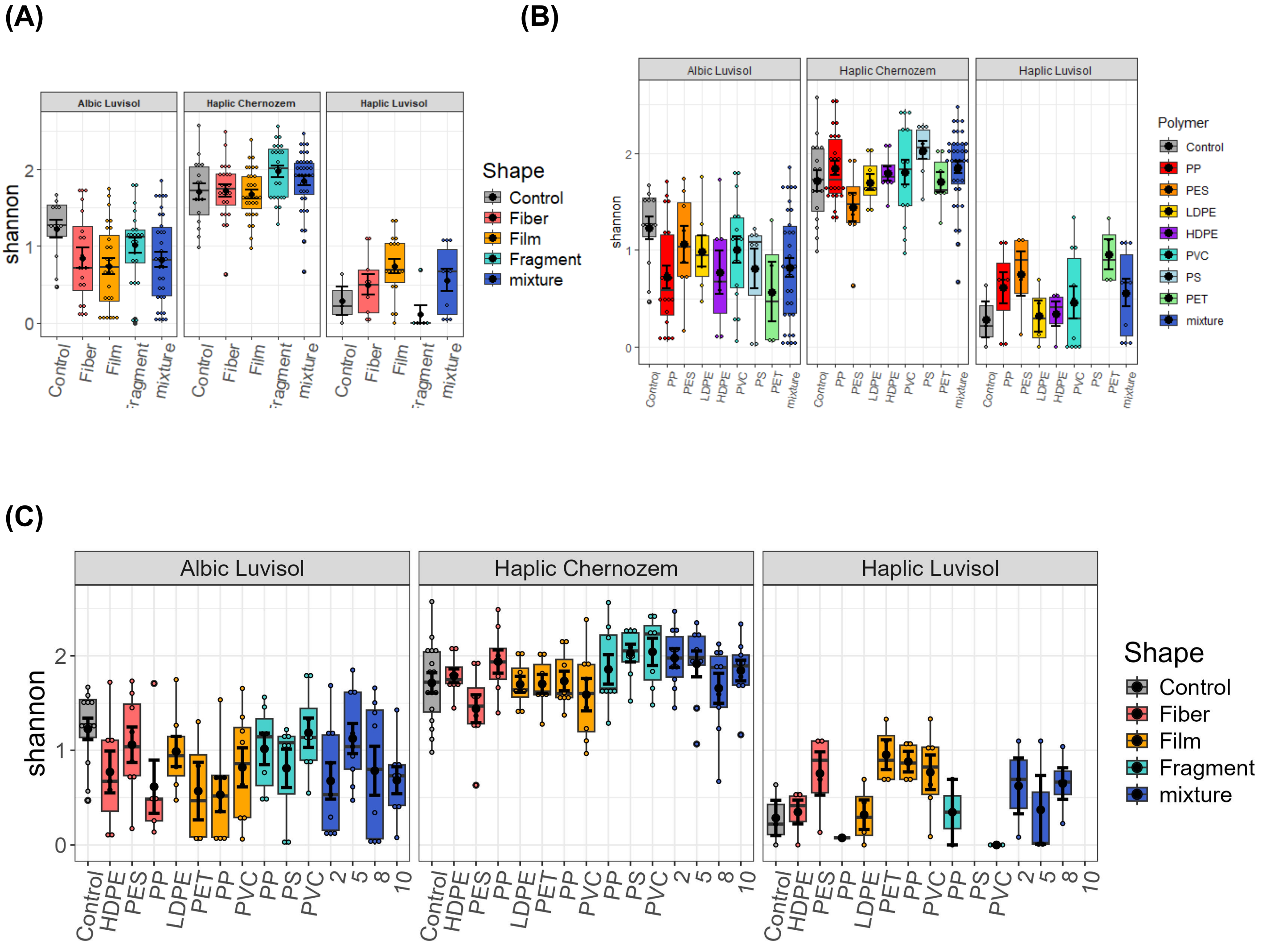

(D)

Albic Luvisol			*Haplic Chernozem*			*Haplic Luvisol*		
**One-way ANOVA**	Treatment	***p*=0.12**	**One-way ANOVA**	Treatment	***p*=0.06**	**One-way ANOVA**	Treatment	***p*=0.07**
**Two-way ANOVA**	Polymer	***p*=0.07**	**Two-way ANOVA**	Polymer	***p*=0.06**	**Two-way ANOVA**	Polymer	***p*=0.07**
	Shape	***p*=0.27**		Shape	***p*=0.24**		Shape	***p*=0.06**
	P:S	***p*=0.75**		P:S	***p*=0.22**		P:S	***p*=0.56**

S. Fig. 3 Panel depicting Shannon diversity index of AMF OTUs in each sample, averaged by micro-plastic shape (**A**), polymer type (**B**) and all combinations labeled by polymer and colored by shape (**C**), and the analysis of variance results (**D**). Black dots are average, error bars are standard error, and gray dots are each individual replicates. Polymers: polypropylene (PP), polyester (PES), high density polyethylene (HDPE), polyethylene terephthalate (PET), low density polyethylene (LDPE), polyvinyl chloride (PVC), and polystyrene (PS). The level 2, 5, 8, and 10 refer to mixtures of microplastics (for details see the Methods section).

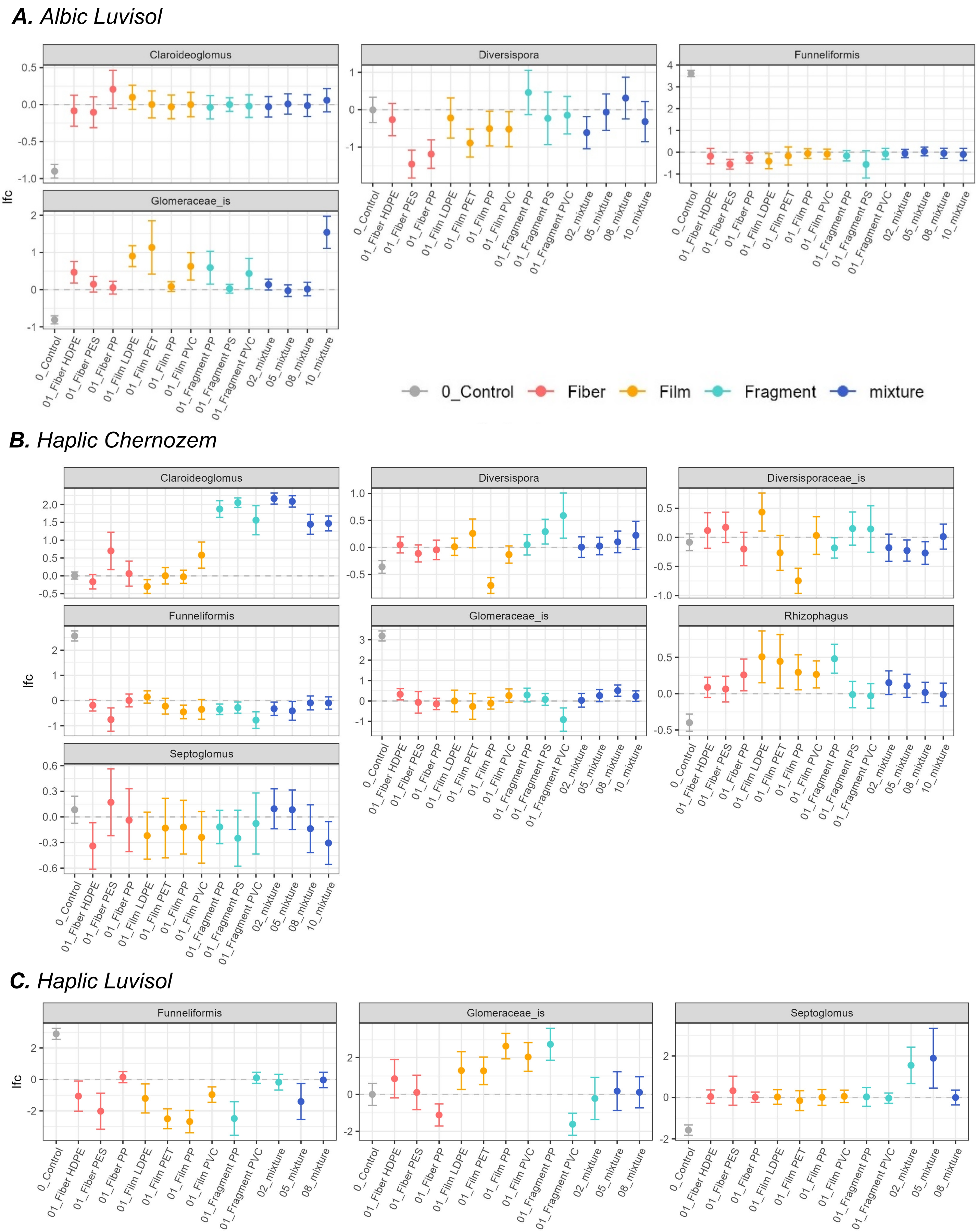

S. Fig. 4 Panel depicting the log fold change (lfc) of AMF genera according to soil types, Albic Luvisol (A.), Haplic Chernozem (B), and Haplic Luvisol (C). Dots are average, error bars are standard error, and asterisk indicate statistical difference in relation to the baseline (ANCOM-BC test). Polymers: polypropylene (PP), polyester (PES), high density polyethylene (HDPE), polyethylene terephthalate (PET), low density polyethylene (LDPE), polyvinyl chloride (PVC), and polystyrene (PS). The level 2, 5, 8, and 10 refer to mixtures of microplastics (for details see the Methods section).

S. Table 1 Details of the three agricultural soils (adapted with permission from Chen et al. 2024).

Soil Type	Sand (%)	Silt (%)	Clay (%)	Bulk density (g/cm^3)	Organic Carbon (g/kg)	Total Nitrogen (g/kg)	pH
Albic Luvisol	73	23	4	1.36	7.0	0.7	5.94
Haplic Chernozem	11	68	21	1.07	20.6	1.8	7.24
Haplic Luvisol	8	77	15	1.16	10	1.0	6.82

S. Table 2 Details of the micro plastics characteristics (adapted with permission from Chen et al. 2024).

Polymer	Shape	Diameter (μm)	Length (mm)	Thickness (mm)	Area (mm^2)
PP	Fiber	30-32	3.00	-	-
PES	Fiber	13	4.00	-	-
HDPE	Fiber	30	1.65	-	-
PET	Film	-	2.49	-	2.71
PP	Film	-	2.41	-	2.66
LDPE	Film	-	2.06	-	2.04
PVC	Film	-	3.63	-	5.95
PP	Fragment	-	1.79	0.5	2.89
PVC	Fragment	-	3.20	0.3	3.88
PS	Fragment	-	1.44	0.5	1.28

Polymers abbreviation: polypropylene (PP), polyester (PES), high density polyethylene (HDPE), polyethylene terephthalate (PET), low density polyethylene (LDPE), polyvinyl chloride (PVC), and polystyrene (PS).

Chen H, Ingraffia R, Schloter M, Brüggemann N, Rillig MC (2024) Effects of multiple microplastic types on growth of winter wheat and soil properties vary in different agricultural soils. Plants People Planet. https://doi.org/10.1002/ppp3.10573
